# The Anatomy of Change: A Scoping Review of Surgical Curriculum Renewal Processes

**DOI:** 10.5334/pme.2010

**Published:** 2025-12-05

**Authors:** Marc A. Seifman, Robyn Woodward-Kron, Roi Y. Kagan, Kirsten Dalrymple, Aimee K. Gardner, Ian Incoll, Lars Konge, John T. Paige, Debra Nestel

**Affiliations:** 1 Department of Surgery (Austin Precinct), The University of Melbourne, Melbourne, Australia; 2 Department of Surgery, Monash Health, Melbourne, Australia; 3 Department of Medical Education, The University of Melbourne, Melbourne, Australia; 4 Department of Surgery and Cancer, Imperial College London, London, United Kingdom; 5 University of Colorado, Colorado, USA; 6 Faculty of Health and Medicine, University of Newcastle, Newcastle, Australia; 7 Copenhagen Academy of Medical Education and Simulation (CAMES), Center for Human Resources and Education, The Capital Region of Denmark, Copenhagen, Denmark; 8 Faculty of Health and Medical Sciences, University of Copenhagen, Copenhagen, Denmark; 9 Department of Surgery, LSU Health New Orleans School of Medicine, New Orleans, USA; 10 School of Clinical, Sciences, Monash University, Clayton, Australia

## Abstract

**Purpose:**

Renewal of surgical curricula is critical in maintaining relevance to contemporary healthcare needs. A curriculum document usually describes its purpose, intended outcomes, content, methods, and assessment and evaluation processes. The document may also consider cultural, professional, political, and social contexts. While there are published curriculum development approaches, guidance on renewal processes is limited. This scoping review aims to synthesise literature on surgical curriculum renewal, to understand the key elements of this process, and identify areas for improvement.

**Methods:**

A scoping review was conducted by the core author team, with co-creation involving a Knowledge User Group (KUG). Seven databases were searched for sources published since 2003. Sources relating to curriculum renewal were included, and data were extracted via iterative and consultative processes involving both core authors and the KUG. Themes were identified via qualitative content analysis and thematic mapping of reported features.

**Results:**

Eighteen sources were included from an originally identified 2359 articles. Six models of curriculum development were characterised, yet no curriculum renewal model was delineated. Terminology was inconsistent. Primary participants (trainees and trainers) tended to be consulted but not included in curriculum renewal teams. Factors including participant engagement, educational support, and financial resources were identified as enablers in particular environments, and considered as barriers in other contexts. Drivers for renewal included changes in surgical education and training; in surgical practice; and, participant concerns.

**Discussion:**

Addressing identified barriers can transform them into catalysts for change. Greater standardisation of terminology in surgical curriculum renewal is needed. The field would benefit from purpose-built frameworks, educational scholarship, co-design, and the implementation of strategies to ensure barriers to renewal become enablers of the surgical curriculum renewal process.

## Introduction

A curriculum is a written document that explicitly describes what is required of learners in terms of the knowledge, skills, and attitudes necessary for clinical practice [1]. It also outlines the methods for supporting learning and assessment [2]. Traditionally, a curriculum resembled a syllabus of content to be covered [3]. More recently, there is a greater holistic appreciation of its position as a philosophy comprising values and beliefs about knowledge content, and methods of imparting and acquiring that knowledge [2]. A curriculum encompasses aims, learning outcomes, teaching and assessment methods [4]; it also describes faculty requirements, supervision, feedback processes, entry requirements, and program organisation [3]. A curriculum should be relevant to the cultural, professional, political, and social environment [5]. Therefore, surgical curricula should consider participants across multiple societal levels, including those who benefit from surgical training and those who gain value from trainees’ patient care [6].

Renewal of curricula is important in maintaining relevance to contemporary healthcare needs and technological advances. Historically, surgical education involved trainees as apprentices under master surgeons [7], with little formalised training. However, the healthcare environment is changing, with rapidly evolving surgical techniques, technological advancements, work-hour restrictions, and changing healthcare delivery models [8]. Concurrently, surgical education has become more regulated, with the introduction of formalised postgraduate education programs [9] and surgical education standards [10]. The practice of surgical education has also become professionalised, reflected in the establishment of the Academy of Surgical Educators in Australia [11], the Academy of Master Surgeon Educators in the United States [12], and the Faculty of Surgical Trainers in the United Kingdom [13]. The constant flux in disease presentations and socio-political contexts [14] may render once state-of-the-art curricula obsolete. Concerns have been raised regarding suboptimal patient outcomes [15] and failures of a previously revered medical system [16]. These challenges necessitate regular review and renewal of surgical curricula to ensure they remain relevant, evidence-based, and effective in producing competent surgical practitioners [14].

Curriculum development requires a methodical approach that incorporates multiple interconnected components. An effective developmental framework must comprehensively address key curricular elements, among which are the knowledge base to be transmitted, appropriate pedagogical methodologies, optimal structural arrangements for learning and instruction, and comprehensive evaluation strategies [17]. Examples of frameworks include Carraccio et al.’s approach to curriculum design [18], Lee et al.’s framework for curriculum development [19], and Kern’s six-step approach to curriculum development [20]. While numerous methods for curriculum development have been published, none explicitly address curriculum renewal, leaving it unclear how program teams engage in this process.

### Stakeholders and participants

Appreciating that surgical education has an impact upon “multiple levels, from the learner to the team, the institution, and the health care system” [21], individuals and organisations may be stratified by their proximity to program implementation. Given the negative connotations of the term *stakeholder* [22,23], we have adopted the term *participant* as an alternative. For the purposes of this article, primary participants include trainees and trainers who directly engage with curricular content. Secondary participants encompass subspecialty societies, educational institutions, clinical competency committees, and program directors who shape and oversee curriculum delivery. Tertiary participants comprise government bodies, accrediting organisations, and community representatives who influence curriculum through broader policy and accountability mechanisms.

### Curriculum design, development, renewal and reform

In the literature, the terminology used to describe curricula and the processes investigated is inconsistent. In general, curriculum *design* is considered as the structure or organisation of the curricular elements, whereas curriculum *development* refers to the processes necessary to produce, execute, and evaluate the curriculum [24]. Some authors discuss steps involved in curriculum design [3], which might be considered similar to the process of curriculum development, and some authors consider development incorporating implementation as well [20]. In this paper, *design* is used to describe the organisation of a curriculum, and *development* is the process of producing the curriculum. Curriculum *renewal* indicates updating existing curricula, often for purposes of accreditation or after a certain amount of time has elapsed, whereas *reform* is suggestive of significant structural change in existing curricula. Where authors have used specific terms, we acknowledge this with italicised text. Inconsistent terminology can result in difficulties with communication and common understanding of curriculum renewal processes.

### Rationale

The critical role of surgical curricula within contemporary surgical education and their influence on the preparation of future surgeons necessitates careful scrutiny of whether renewal initiatives are grounded in empirical evidence and/or informed by established theoretical frameworks. For example, as many competency-based medical education (CBME) curricula are currently undergoing renewal or approaching re-accreditation, a comprehensive examination of renewal processes is warranted. This scoping review aims to synthesise and comprehensively review published literature [25] on surgical curriculum renewal, to understand the key elements of this process, and identify potential areas for improvement [26].

## Methods

Using the five-step framework of Arksey and O’Malley (2005) [27] and incorporating recommendations from Levac et al. (2010) [28], we undertook a scoping review of surgical curriculum renewal. In addition to the core author team (MS, RWK, RK, DN), we established a Knowledge User Group (KUG) (KD, AG, II, LK, JP) to increase the research relevance, transparency and rigor through the adoption of the co-creation through consultation model [29].

### Step 1: Identifying the research questions

Our overarching research question was: “What is known about surgical curriculum renewal?”

Using elements of the who, what, when, where, why, and how framework [30,31], specific questions included:

1. Who is involved in renewing surgical curricula?
2. What are enablers and barriers of surgical curriculum renewal?
3. Why (and when) are curricula renewed: What are the drivers for surgical curriculum renewal?
4. How are curricula renewed: Which frameworks are used in surgical curriculum renewal?

### Step 2: Identifying relevant sources

In conjunction with a health network librarian, a search strategy was developed with the intention of capturing maximal results, which could then be refined. We used the Population, Context, Concept (PCC) framework [25], to develop the search strategy. This strategy was modified to employ search terms appropriate for each database. After piloting, alterations were made to optimise the strategy (Appendix 1). Evidence resources included electronic databases, bibliographic lists of identified journal sources, citation searching, and hand-searching of key journals. In this paper, *source* is used to denote a publication identified via these searches.

The search was conducted in March 2024. To maintain currency, a further search was repeated in October 2024. Sources were identified in three Ovid MEDLINE databases (MEDLINE, In-Process & Other Non-Indexed Citations, and Epub Ahead of Print), Embase, Scopus, Educational Resources Information Centre (ERIC), and PsychINFO. There were no qualifications on language or publication type employed. Sources published prior to 2003 were excluded, as the introduction of CBME has occurred mainly since then.

Endnote X9 (Clarivate Analytics, Philadelphia, Pennsylvania) was used to export sources to individual reference libraries reflecting the origin of sources. The references were uploaded to the Covidence online platform (Veritas Health Innovation Ltd, Melbourne, Australia). The reference libraries were subsequently combined, and duplicate references removed. A search of the grey literature was not conducted given the volume of sources identified from existing databases and the subsequent bibliographic and citation searches.

### Step 3: Source selection

Title and abstract screening was undertaken independently by two reviewers (MS, RK), and conflicts reviewed until consensus was reached. Initial inclusion and exclusion criteria were applied to the decision-making process (Appendix 2), and throughout the source selection process, an iterative process of modifications to these criteria occurred [28]. Full text review was undertaken in the same manner, and conflicts that could not be resolved were referred to a third reviewer (DN) for resolution. The resultant sources underwent reference and citation searches, following which the above process of title and abstract screening, and full text review, was conducted. This process was continued until no further sources were identified via reference and citation searching.

### Step 4: Charting the data

Variables aligned with the research questions were defined and used to populate a data extraction template in Covidence. Demographics of the sources were incorporated into the template, including year and country of publication, and source publication in an education-focused journal [26,32]. We extracted information with reference to educational theories and renewal frameworks, who was involved in renewal, and documented barriers and enablers of curriculum renewal. Recent literature examining curriculum-focused research has identified, amongst other issues, concerns regarding “conceptual obscurity” – failure to employ relevant conceptual frameworks to curriculum research [33]. We therefore used Kern’s curriculum development framework [20] to systematically identify steps outlined in the renewal process. The framework was selected for its simplicity in approach and relevance to surgical curricula, having been developed for medical contexts [3] (Box 1).

**Box 1 Frameworks which may be used for curricula renewal.**

**Table:** 


**Framework**	**Steps**

Kern [20]	Problem Identification and General Needs Assessment
	Targeted Needs Assessment
	Goals and Objectives
	Educational Strategies
	Implementation
	Evaluation and Feedback

Lee et al. [19]	Big picture decisions: the why?
	Defining capabilities of graduates: the what?
	Teaching, learning and assessment: the how?
	Organisation: the where?

Carraccio et al. [18]	Competency identification
	Determination of competency components and performance levels
	Competency evaluation
	Overall assessment of the process

Knox et al. [54]	Identify specifically what is expected of graduating plastic surgery residents (competencies)
	Break down these competencies into a logical stepwise series of markers of ability (milestones)
	Identify or develop educational strategies to facilitate instruction of these skills
	Develop assessment tools that are easy to use and yet capable of accurately placing trainees along this continuum of achievement
	Perform an outcomes evaluation to determine the effectiveness of competency-based medical education for achieving competencies and provide feedback for program improvement

Frank et al. [55]	Identify the abilities needed of graduates
	Explicitly define the required competencies and their components
	Define milestones along a development path for competencies
	Select educational activities, experiences and instructional methods
	Select assessment tools to measure progress along the milestones
	Design an outcomes evaluation of the program

ADDIE	Analysis
	Design
	Development
	Implementation
	Evaluation

All sources underwent data extraction by two independent reviewers: one author (MS) extracted data from all sources, and three authors (RK, DN, RWK) extracted data from selected sources, following which a consultative process was undertaken to ensure accuracy of charting.

### Step 5: Collating, summarising, and reporting the results

Extracted data were exported to Microsoft Excel (Microsoft Excel for Mac, 2020, Microsoft Corporation, Redmond, Washington) for analysis. Descriptive statistics were performed on demographic data. Qualitative content analysis [34] was employed in identifying the steps employed in curriculum renewal (through the lens of Kern’s steps for curriculum development [20]). Inductive thematic mapping of reported features was used to examine the drivers, as well as enablers and barriers, of curriculum renewal. Deductive thematic mapping of reported features using an adaptation of stakeholder theory [35] was employed to examine those involved in curriculum renewal.

### Knowledge user group consultation

The KUG was comprised of five international surgical education experts with relevant leadership and curriculum responsibilities. They also work in different countries – Australia, Denmark, UK and the USA. The KUG was consulted on the research questions (Step 1); study design, suggestions regarding renewal, additional sources to be included in screening (Step 2); advice on terminology, the data extraction template (Step 4); and interpretation of summarised data and reporting the results (Step 5).

## Results

**Figure 1:**
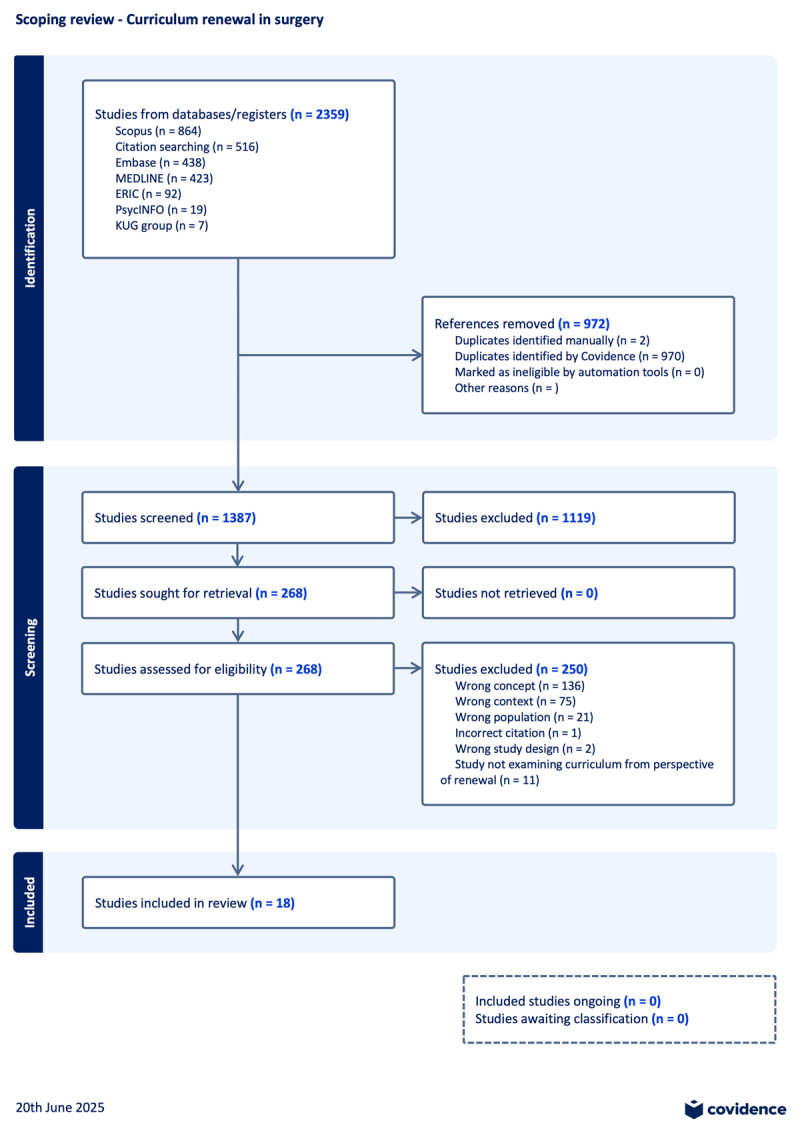
PRISMA flowchart depicting source search and selection process. (alt = ”PRISMA flowchart”).

The selection process was mapped using a PRISMA flowchart (Figure 1). From the initial search, 1836 sources were identified, from which duplicates were removed. The identified sources underwent the same screening process until no further results were generated; three rounds of citation and bibliographic searches occurred. In total, 2359 sources were identified following which 972 duplicates were removed. Of the 1387 sources that underwent title and abstract screening, and of the 268 sources that underwent full text review, 18 sources met final inclusion criteria (Appendix 3).

### Source characteristics

Sources were published between 2007 and 2023, with the majority (n = 14) [36,37,38,39,40,41,42,43,44,45,46,47,48,49] originating from North America (Table 1). Surgical subspecialties addressed in the sources, included general surgery (n = 7) [36,37,39,41,45,46,49], neurosurgery (n = 4) [40,42,50,51], orthopaedic surgery (n = 3) [43,47,52], and plastic and reconstructive surgery (n = 1) [48]. Study research methods included surveys, individual interviews, and focus groups (n = 8) [39,41,42,44,45,48,50,53], articles detailing reflections on experiences (n = 4) [36,43,46,52], literature reviews (n = 2) [40,51], summative and formative assessments of trainee performance (n = 2) [37,47], and a Delphi study (n = 1) [49].

**Table 1:** Characteristics of included sources.


CHARACTE RISTICS	SOURCE																	

	SACHDEVA 2007 [36]	LILLEVANG 2009 [53]	WEBB 2009 [37]	MOALEM 2012 [38]	TAPIA 2014 [39]	MANSOURI 2015 [40]	DROLET 2017 [41]	CON FORTI 2018 [42]	NOUSIAINEN 2018 [43]	GARBARINO 2019 [44]	DICKINSON 2020 [46]	GADJRADJ 2020 [50]	INCOLL 2020 [52]	MADOR 2020 [45]	CHAN 2021 [47]	DADA 2022 [51]	KEARNEY 2022 [48]	RYAN 2024 [49]

**Region study undertaken**																		

North America (n = 14)	X		X	X	X	X	X	X	X	X	X			X	X		X	X

Europe (n = 2)		X										X						

Africa (n = 1)																X		

Australia (n = 1)													X					

**Education-focused journal**																		

Yes (n = 8)		X		X			X	X	X					X	X			X

**Open access source**																		

Yes (n = 8)	X	X	X	X	X							X		X	X			

**Surgical subspecialty**																		

All surgery (n = 2)		X		X														

General surgery (n = 7)	X		X		X		X				X			X				X

Neurosurgery (n = 4)						X		X				X				X		

Orthopaedic surgery (n = 3)									X				X		X			

Plastic and Reconstructive surgery (n = 1)																	X	

Obstetrics/Gynaecology (n = 1)										X								

**Research methods**																		

Assessment tools (n = 2)			X												X			

Delphi study (n = 1)																		X

Interviews/Focus groups (n = 3)		X						X						X				

Literature review (n = 2)						X										X		

Reflection on experience (n = 4)	X								X		X		X					

Survey/Questionnaire (n = 7)		X			X		X	X		X		X					X	

**Educational theory referenced**																		

Yes (n = 1)											X							

**Curriculum development framework referenced**																		

Carraccio et al. [18] (n = 1)						X												

Kern [20] (n = 3)					X						X			X				

Knox et al. [54] (n = 1)						X												

Lee et al. [19] (n = 1)														X				

**Evidence of the use of the steps in Kern’s framework**																		

Step 1: Problem identification and general needs assessment (n = 9)				X	X	X				X	X		X	X	X	X		

Step 2: Targeted needs assessment (n = 7)			X		X						X	X	X	X	X			

Step 3: Goals and objectives (n = 9)	X			X	X				X		X	X	X	X				X

Step 4: Educational strategies (n = 7)					X	X			X	X	X		X					X

Step 5: Implementation (n = 5)		X			X				X		X		X					

Step 6: Evaluation and feedback (n = 12)	X	X	X				X	X	X	X	X	X			X	X	X	


Note: Assessment tools include: MCQ: multiple choice question examination; ABSITE: American Board of Surgery In-training Examination; OSATS: Objective Structured Assessment of Technical Skill; UM-GCDMA: University of Michigan Geriatrics Clinical Decision-Making Assessment; UCLA Geriatrics Attitudes Scale; ITERs: In-Training Evaluation Reports; RES: Rotation Effectiveness Scores.

Ten sources [36,38,41,42,43,44,45,47,49,53] originated from education-focused journals, whereas eight originated from clinically-focused journals [37,39,40,46,48,50,51,52]. One source [46] referenced an educational concept (Bloom’s taxonomy).

### Who is involved in surgical curriculum renewal?

#### Curriculum renewal team

The representation of entities across different levels within the curriculum renewal team was examined (Table 2). Secondary participant level representation was most prevalent (n = 9) [36,37,39,42,43,47,49,52,53], followed by tertiary participant level (n = 4) [36,42,49,53] and primary participant level (n = 3) [39,49,50]. Five sources incorporated multiple levels of representation [36,39,42,49,53]. One source included participants at all levels [49].

**Table 2:** Composition of curriculum renewal team by participant level.


LEVEL OF PARTICIPANT	DETAILS	SOURCES

Primary	Trainees, trainers/surgeons	[39,49,50]

Secondary	Subspecialty organisations Surgical education organisations Hospital boards/committees University committees Program directors Clinical competency committees	[36,37,39,42,43,47,49,52,53]

Tertiary	Accrediting/regulatory bodies National surgical colleges	[36,42,49,53]

Unclear		[38,40,41,44,45,46,48,51]

Combination: Primary-Secondary		[39]

Combination: Secondary-Tertiary		[36,42,53]

Combination: All levels		[49]


#### Participant consultation

Regarding participant consultation during curriculum renewal (Table 3), the distribution differed from team composition. Primary participants were most frequently consulted (n = 10) [37,39,42,45,46,47,49,50,51,52], followed by secondary participants (n = 8) [41,42,44,45,48,49,52,53], and tertiary participants (n = 2) [49,53]. Participant consultation across all levels was reported in one source [49].

**Table 3:** Participant involvement by level.


LEVEL OF PARTICIPANT	DETAILS	SOURCES

Primary	Trainees, trainers/surgeons	[37,39,42,45,46,47,49,50,51,52]

Secondary	Subspecialty organisations Surgical education organisations Hospital boards/committees University committees Program directors Clinical competency committees	[41,42,44,45,48,49,52,53]

Tertiary	Accrediting/regulatory bodies National surgical colleges	[49,53]

Unclear/Not applicable		[36,38,40,43]

Combination: Primary-Secondary		[42,45,52]

Combination: Secondary-Tertiary		[53]

Combination: All levels		[49]


### What are enablers and barriers of surgical curriculum renewal?

**Figure 2:**
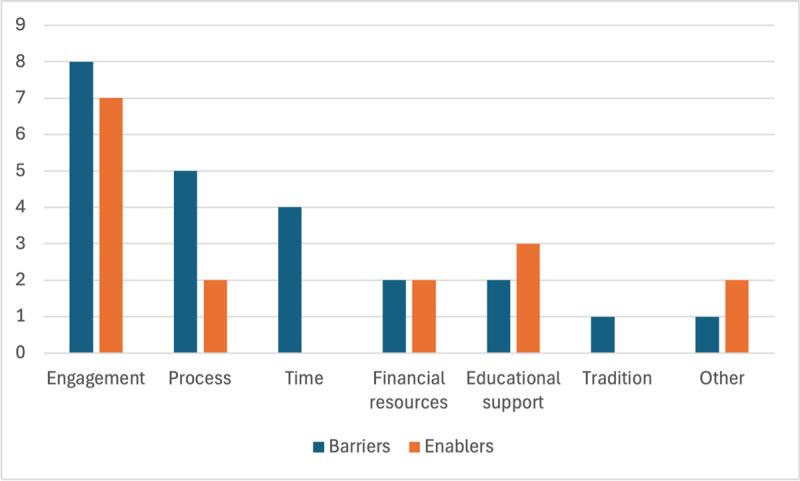
Barriers and Enablers of Surgical Curriculum Renewal (alt = ” Barriers and Enablers of Surgical Curriculum Renewal”).

Certain factors identified as enablers in particular environments were considered barriers in others and vice versa. Engagement of participants was a prominent enabler of the process (n = 7) [39,41,43,48,49,50,52]. Educational support in the form of seminars supporting the development process and educational organisational motivation for the process were viewed positively (n = 3) [37,52,53]. Process conduct and design including clear guideline development and rigorous research methodology (n = 2) [44,53], as well as sufficient financial resources (n = 2) [47,52], were considered enablers of the process. Additional themes of learner satisfaction (n = 1) [46] and perceived value of the renewed curriculum (n = 1) [42] were identified as enablers (Figure 2). Enablers were not reported in six sources.

Barriers included poor participant representation (n = 8) [37,41,42,43,44,45,47,50], issues with the conduct and design of the renewal process (n = 5) [40,44,47,50,53], lack of resources concerning time (n = 4) [37,43,46,53] and finances (n = 2) [43,46], poor educational support concerning faculty development and pedagogical support (n = 2) [43,53], and tradition (n = 1) [42] (Figure 2). Barriers were not reported in seven sources.

### What are the drivers for surgical curriculum renewal?

Three themes were identified as drivers for renewal: (1) Changes in surgical education and training; (2) Changes in surgical practice; and (3) Participant concerns.

#### Changes in surgical education and training

Changes in the conduct and delivery of surgical education and training were reported as catalysts for curriculum renewal. These drivers included decreased clinical exposure and time for educational activities due to work hour restrictions [37], and the diminished time and autonomy afforded trainees due to increases in regulation and requirements for increased efficiency in patient care delivery [36]. Other changes included the challenges surrounding the introduction of a competency-based curriculum: these included ambiguity surrounding curricular objectives [47] and insufficient resources [41] required to accommodate increased accreditation and regulatory requirements [37,44]. Additionally, the increasing role (and, in some instances, the mandating) of simulation in surgical education and training [36,46] prompted curriculum renewal.

#### Changes in surgical practice

Similar to changes in education and training, decreased clinical and operative exposure for trainees [36,49] was reported, attributed to more outpatient care, a decreased length of stay within the hospital system [36], a preference for non-operative management where possible [45,50], and increased certification and regulatory requirements [39]. Renewal was also justified due to concerns voiced regarding the ability of existing curricula to adapt to increasing patient complexity [45], technological advances [38], and an increasing body of medical knowledge [40]. Additionally, there are concerns regarding the impact of work hour restrictions on clinical experience [37], involving “loss of continuity of care, development of a shift-worker mentality, decrease in the sense of resident ownership of patient care, and the potential risk to patient safety resulting from frequent transfers of care” [36]. These concerns have resulted in a drive for curriculum renewal.

#### Participant concerns

At the primary participant level, both trainees and trainers shared similar concerns, including insufficient educational quality [39,47,53], and workforce physical and mental well-being [45,50]. At the secondary participant level, specialty societies involved in the examination and certification of trainees have questioned the adequacy of current training methods [44], particularly regarding evaluation quality and individualisation in training [38]. At the tertiary level (government bodies, accrediting organisations, and community representatives), participants highlight issues with training quality [45,52,53], public concerns regarding safety and quality-of-care [36,45], and challenges posed by socio-political contexts including migration patterns [51], political considerations [53], geographical barriers in remote areas [45,47,49], and healthcare system needs [42] as factors prompting curriculum renewal. Workforce recruitment and retention problems [51,53] are also significant. These multi-level concerns collectively drive the need for curriculum renewal.

### Which frameworks are used in surgical curriculum renewal?

Frameworks for curriculum design or development were referenced in four sources. Kern’s [20] six-step approach to curriculum development was referenced in three sources [39,45,46]. One source noted Carraccio et al.’s [18] four-step approach to curriculum design [40]. Knox et al.’s [54] five-step approach to planning a CBME curriculum for plastic surgery was mentioned once [40], though it should be noted that their framework was adapted from the six steps proposed by Frank et al. [55] in planning CBME curricula. Lee et al.’s [19] four-dimensional curriculum development framework was mentioned once [45]. Additionally, the ADDIE framework of instructional systems development, the precise origins of which are unclear [56], was suggested by the KUG (Box 1). Fourteen sources [36,37,38,41,42,43,44,47,48,49,50,51,52,53] did not explicitly mention a curriculum design, development, or renewal framework.

Kern’s framework [20] was used to determine which processes were employed implicitly in the selected sources (Table 1). The stage of ‘Problem Identification and General Needs Assessment’ was employed by nine sources [38,39,40,44,45,46,47,51,52], ‘Targeted Needs Assessment’ by seven sources [37,39,45,46,47,50,52], ‘Goals and Objectives’ by nine sources [36,38,39,43,45,46,49,50,52], ‘Educational Strategies’ by seven sources [39,40,43,44,46,49,52], ‘Implementation’ by five sources [39,43,46,52,53], and ‘Evaluation and Feedback’ was employed by 12 sources [36,37,41,42,43,44,46,47,48,50,51,53]. To determine how comprehensive the selected sources were in their employment of the entire curriculum renewal process, we examined how many of Kern’s (2015) [20] six steps were used by the selected sources. Fourteen sources [36,37,38,40,41,42,44,45,47,48,49,50,51,53] used fewer than four steps, seven sources [36,37,38,40,49,51,53] used two of the steps, and one [46] used all six steps of the curriculum development framework.

## Discussion

The key findings in this scoping review are:

– A tendency for a top-down approach to team composition in curriculum renewal exists;
– Factors in the renewal process which may act as either enablers or barriers, depending upon their context;
– The factors driving renewal include changes in both surgical education and training and surgical practice as well as participant concerns; and,
– Existing curriculum development frameworks are rarely explicitly referenced.

In these next sections, we discuss the findings, consider their implications for curriculum renewal, and propose potential opportunities for improving this process.

### Involvement in surgical curriculum renewal

Surgical curriculum renewal processes are inherently embedded within distinct sociopolitical frameworks that vary according to institutional environment, governance architecture, accreditation mandates, and involvement of participants. Compliance with jurisdiction-specific regulatory requirements represents a fundamental prerequisite for accreditation, necessitating integration of prescribed competency standards including procedural volume thresholds, research obligations, mandatory foundational coursework, and successful examination completion. Accordingly, curricula must consider and incorporate these requirements throughout curriculum renewal. Appreciating this regulatory complexity, this study sought to explore the distribution of roles of those involved in surgical curriculum renewal – both those directly involved in renewing the curriculum, and those consulted as part of the process.

Our findings indicate that curriculum renewal is generally conducted by those further removed from curriculum implementation, potentially indicating that learning objectives and experiences are determined by mandating authorities. The formulation of surgical curricula has widespread impact, influencing all societal levels. It would therefore stand to reason that a wide representation of participants from all societal levels would be engaged in all aspects of the curriculum renewal process – both in the curriculum renewal team and in the participants consulted in this process. Despite the benefits of addressing curriculum problems via participant involvement having been recognised for decades [57], this process is not routinely undertaken. Those somewhat removed from the detailed implementation of curricula may possess a broader perspective on curricular goals and context, yet this distance may render them unaware of the practical considerations relevant to the trainee learning experience [58,59]. *Co-design*, at times synonymous with co-creation and co-participation, is increasingly being recognised for its value in health professions education [60]. In contrast to the trainee being a passive beneficiary of a curriculum, this active partnership is “a collaborative, reciprocal process through which all participants have the opportunity to contribute equally, although not necessarily in the same ways, to curricular or pedagogical conceptualisation, decision making, implementation, investigation, or analysis” [61]. The involvement of those with an interest in the curriculum renewal process is imperative, as it fosters beneficial effects on the psychosocial learning environment, motivation and metacognition, and the quality of educational design [62].

Additionally, the tertiary-level participants may not necessarily have a grounding in surgical education practice [59] or might not consider the role of those with such a background valuable in the renewal process. However, without expert contribution, curriculum renewal teams may fail to incorporate relevant evidence-based educational principles. Appropriate representation of educational experts and those at all sociological levels on curriculum renewal teams would improve the practicality and relevance of the curriculum, and also promote commitment from these participants, resulting in enhanced student learning and empowerment as agents of sustainable educational change [60].

### Enablers and barriers of surgical curriculum renewal

Curriculum renewal is influenced by a range of contextual factors that may function either as enablers or barriers to the process. Recognising and understanding this duality is essential for the development of effective renewal strategies. The absence of institutional support and resources can significantly impede progress, whereas their presence facilitates and accelerates curriculum transformation. Pedagogical support similarly exhibits this dichotomy; institutions that lack educational expertise may hinder renewal efforts, while those that offer educational development support and access to advisors from accrediting bodies can enhance the process [53].

The success of surgical curriculum renewal is particularly contingent upon the commitment and awareness of those leading the initiative. When curriculum leaders are attuned to these dynamic factors, they are better positioned to strategically convert potential obstacles into facilitators through targeted adjustments in institutional practices and resource distribution. For instance, financial support serves as a critical enabler when sufficient, allowing for the allocation of necessary resources and personnel; conversely, inadequate funding may act as a significant barrier. Similarly, the provision of educational resources – such as seminars, materials, and structured support – can promote renewal, whereas their absence may discourage engagement.

A transformation of barriers into enablers of curriculum renewal requires not only motivation but also deliberate action to cultivate conditions conducive to meaningful change. Although features of successful curricular change have been described in other educational contexts [63], there remains a paucity of literature addressing these features within surgical education. For curriculum renewal to be effectively implemented, leaders must not only identify and understand these multifaceted influences but also develop targeted strategies to leverage them as catalysts for change. In doing so, they can foster an environment that supports sustained and impactful educational transformation.

### Drivers of curriculum renewal

The evolution of surgical practice and training landscapes has rendered many existing curricula obsolete. Training opportunities have been significantly diminished through the convergence of healthcare fiscal constraints, regulatory work-hour limitations, and the progressive migration toward ambulatory care models [64]. These systemic changes have generated substantive concerns among educational participants regarding programmatic efficacy and outcomes. The three themes identified – transformations in surgical education and training paradigms, evolution of surgical practice modalities, and participant concerns – demonstrate direct concordance with the fundamental objectives of prescriptive curriculum design frameworks, namely that educational programs must serve the requirements of their contextual socio-political environments. The prioritization of these considerations throughout the renewal process can effectively address participant concerns while ensuring curricular relevance and contemporary applicability.

A key development that has resulted from the above concerns has been the implementation of CBME in recent decades. This educational paradigm has altered both the conduct and pedagogy of surgical practice [65]. The requirement to achieve specific milestones rather than a time-based program with scheduled assessment points [55] has radically changed the trainee experience. These modifications to surgical training have sought to address the concerns regarding curricula losing currency. Rather than functioning solely as an outcome of curriculum renewal processes, CBME has simultaneously served as a catalyst for such initiatives. The CBME phenomenon exemplifies the cyclical nature of curriculum development and renewal processes, as articulated by Kern [20], wherein the evaluation of existing curricula naturally informs needs assessment activities and subsequent curriculum renewal initiatives.

The ability to educate future surgeons was once regarded an inherent skill of existing surgeons, with little attention given to the theory, structure and practice of surgical education. As consideration of these factors has grown, the volume of surgical education research has increased exponentially [26]. The establishment of dedicated academic societies [11,12,13] and increased scholarly focus demonstrate the emerging science of surgical education. Where proposed modifications to clinical practice demand evidence-based foundations, it follows logically that surgical education should embrace similar standards, with developments in educational science serving as drivers of curriculum renewal.

### Curriculum renewal frameworks

Due to an absence of curriculum renewal frameworks, the findings of this review show that existing curriculum development frameworks are typically repurposed for curriculum renewal. Moreover, only four sources used any framework. It may be that the existing frameworks are difficult to follow, that they might be deficient, or that the process of renewal is so intuitive that a formal framework is not required. It is also possible that those engaged in curriculum renewal either do not see value in the use of frameworks or are unaware of their existence. While the use of different or even a combination of frameworks may be beneficial [66], a widely-accepted curriculum renewal framework may provide guidance for those engaged in surgical curriculum renewal.

The employment of formalised frameworks in guiding the process of curriculum renewal enables the renewal team to consider all aspects of a curriculum. Sources using frameworks did not address all steps of the frameworks employed, with more sources engaging in ‘Evaluation and Feedback,’ than other steps, such as ‘Implementation’ (Table 2). Emphasising ‘Evaluation and Feedback’ can generate valuable data to identify curricular elements that require modification. In contrast to components of curriculum development frameworks, specific evaluation models, such as the Context, Input, Process, Product (CIPP) model [67], offer structured approaches for assessing existing curricula. This might enhance the ‘Evaluation and Feedback’ component of curriculum renewal.

Our findings demonstrate that ‘Implementation’ is the least employed step of Kern’s framework in curriculum renewal. ‘Implementation’ is multifaceted, including “obtaining political support; identifying and procuring resources; identifying and addressing barriers to implementation; introducing the curriculum (e.g., piloting the curriculum for a friendly audience before presenting it to all targeted learners, phasing in the curriculum one part at a time); administering the curriculum; and refining the curriculum over successive cycles” [20]. Of note, Kern highlights that “implementation is critical to the success of a curriculum. It is the step that converts a mental exercise to reality” [20]. ‘Implementation’ requires consistent and prolonged involvement in the curriculum renewal process and may not align with the mandate of the curriculum renewal team to complete its task within a particular time frame. However, without consideration of ‘Implementation’, curricula run the risk of being on a curricular carousel [68], where there is repetition of similar concepts with minimal reform [69]. This may sometimes occur because of the temptation to apply relatively ‘quick fixes’ to complex issues [69] in the complex adaptive system that is a curriculum [70].

### Terminology

This study has identified inconsistencies in the literature in the use of language surrounding curricula, where certain terms might be used interchangeably. Curricula themselves are at times discussed as syllabi, ignoring the wider philosophy and role of curricula. Development, design, and implementation are at times blended, and the degree of change might impact upon whether curricula undergo renewal or reform. The presence of a common language in surgical education and curricula would enhance communication, allow for improved comparison of curricula, and standardise reference to elements of surgical training. Ultimately, it would facilitate the growth of both the study and practice of surgical education.

### Limitations

The search was limited to the last 20 years, the same period in which surgical specialties have had comprehensive curricula change due to initiatives such as the United Kingdom’s Intercollegiate Surgical Curriculum Project [71]. It is possible that the scope of this search was too limited given that curricula are constantly evolving. The exclusion criteria discarded sources relating to non-surgical specialties, despite there being several specialties with significant procedural components that may have otherwise contributed to the scoping review. While sources from all countries were included in the scoping review, the majority originated from North America, potentially resulting in geographic sampling bias. This may impact the findings of this study, as the context of the curriculum renewal is important: there may be countries with more prescriptive professional associations which drive curriculum renewal but do not publish their processes.

Given the absence of established frameworks for systematic curriculum renewal identification, this study adopted Kern’s curriculum development framework as the methodological foundation. This decision was predicated on its simplicity in approach and relevance to surgical curricula, having been developed for medical contexts. However, the adaptation of Kern’s framework from a development to a renewal paradigm may introduce methodological constraints and conceptual limitations. While Kern’s ‘Implementation’ phase (Step 5) addresses critical factors including political support mechanisms, resource allocation, and barrier mitigation strategies [20], the framework is less specific regarding issues that may be more relevant in the setting of renewal compared with development. Notably, this investigation identified institutional tradition as a significant renewal barrier, representing a broader category of legacy constraints that may impede curricular renewal efforts. Further, Kern’s framework does not explicitly incorporate principles from change management theory or established methods for ensuring successful change. Given the absence of purpose-designed curriculum renewal frameworks in the literature, Kern’s model represents a methodologically defensible and contextually appropriate analytical framework available for this investigation.

## Conclusions

This scoping review reveals gaps in surgical curriculum renewal processes, despite the increasing professionalisation of surgical education. Strategically addressing identified barriers can transform them into catalysts for purposeful change. Key drivers for renewal include concern over whether the changing landscapes of surgery and surgical education have rendered curricula obsolete. We did not identify any frameworks specifically designed for curriculum renewal. Consistent and clear use of terminology would enable more meaningful collaboration, comparison and discourse. The field would benefit from purpose-built frameworks, educational scholarship driving change, co-design, and the implementation of strategies to ensure barriers to renewal become enablers of meaningful change.

## Additional File

The additional file for this article can be found as follows:

10.5334/pme.2010.s1 Appendices. Appendix 1 to 3.
