# Supplementary material for: The Anatomy of Change: A Scoping Review of Surgical Curriculum Renewal Processes
Source: Perspect Med Educ. 2025 Dec 5;14(1):956–73. doi: 10.5334/pme.2010 (PMC12679985; doi:10.5334/pme.2010)
Supplement: Appendices. — Appendix 1 to 3. [file pme-14-1-2010-s1.pdf]

Appendix 1 – Search Strategy

ERIC

(descriptor:"Surgery" OR descriptor:"Surgeons General" OR descriptor:"Surgeons" OR descriptor:"Medical education" OR surgery OR surgical OR surgeon*) AND (renewal OR renewed OR revision OR revise OR evaluat* OR review*) AND (descriptor:"Core curriculum" OR descriptor:"Curriculum development" OR descriptor:"Curriculum selection" OR curricul* OR "course content")

Scopus

(surgery OR surgical OR surgeon*) AND (medic* OR cardiothoracic OR cardiac OR thoracic* OR plastic* OR vascular OR urolog* OR otolaryngolog* OR ent* OR ear OR nose OR throat OR orthopaedic* OR orthopedic* OR neurosurg* OR paediatric* OR pediatric* OR obstetric* OR gynaecolog* OR gynecolog* OR ophthalmolog*)

AND

((educat* OR teach* OR training) W/10 (medic* OR surg*))

AND

((educat* OR curricul*) W/5 (renewal OR renewed OR revision OR revise OR evaluat* OR review*))

AND

curricul* OR "course content"

MEDLINE

1 General Surgery/ or exp Surgeons/ or exp Specialties, Surgical/ (237396)
2 (surg* adj5 (cardiothoracic or cardiac or thoracic* or plastic* or vascular or urolog* or otolaryngolog* or ENT* or ear or nose or throat or orthopaedic* or orthopedic* or neurosurg* or paediatric* or pediatric* or obstetric* or gynaecolog* or gynecolog* or ophthalmolog*)).mp. (457938)
3 1 or 2 (626969)
4 education, medical/ or education, medical, continuing/ or education, medical, graduate/ (117088)
5 ((educat* or teach* or training) adj10 (medic* or surg*)).ti,ab,kw. (194251)
6 4 or 5 (266620)
7 ((educat* or curricul*) adj5 (renewal or renewed or revision or revise or evaluat* or review*)).ti,ab,kw. (31014)
8 *Curriculum/ (26980)
9 (curricul* or course content).ti,ab,kw. (78200)
10 8 or 9 (88638)
11 3 and 6 and 7 and 10 (440)
12 limit 11 to yr="2003 -Current" (396)

EMBASE

1 exp General Surgery/ or exp Surgeon/ (219795)
2 (surg* adj5 (cardiothoracic or cardiac or thoracic* or plastic* or vascular or urolog* or otolaryngolog* or ENT* or ear or nose or throat or orthopaedic* or orthopedic* or neurosurg* or paediatric* or pediatric* or obstetric* or gynaecolog* or gynecolog* or ophthalmolog*)).mp. (589075)
3 1 or 2 (746550)

4 medical education/ or continuing education/ or graduate education/ or postgraduate education/ or education program/ (333889)
5 ((educat* or teach* or training) adj10 (medic* or surg*)).ti,ab,kw. (261750)
6 4 or 5 (504410)
7 ((educat* or curricul*) adj5 (renewal or renewed or revision or revise or evaluat* or review*)).ti,ab,kw. (41804)
8 *curriculum/ (29348)
9 (curricul* or course content).ti,ab,kw. (100480)
10 8 or 9 (109225)
11 3 and 6 and 7 and 10 (434)
12 limit 11 to yr="2003 -Current" (416)

PsycINFO

1 exp Surgery/ or exp Surgeons/ (57332)
2 (surg* adj5 (cardiothoracic or cardiac or thoracic* or plastic* or vascular or urolog* or otolaryngolog* or ENT* or ear or nose or throat or orthopaedic* or orthopedic* or neurosurg* or paediatric* or pediatric* or obstetric* or gynaecolog* or gynecolog* or ophthalmolog*)).mp. (6189)
3 1 or 2 (59760)
4 Medical Education/ or Continuing Education/ or Graduate Education/ (25203)
5 ((educat* or teach* or training) adj10 (medic* or surg*)).mp. (52583)
6 4 or 5 (56998)
7 ((educat* or curricul*) adj5 (renewal or renewed or revision or revise or evaluat* or review*)).mp. (31194)
8 Curriculum/ or Curriculum Development/ (37234)
9 (curricul* or course content).mp. (94534)
10 8 or 9 (94534)
11 3 and 6 and 7 and 10 (21)
12 limit 11 to yr="2003 -Current" (19)

Appendix 2 – Inclusion and Exclusion Criteria

Inclusion and exclusion criteria determined via an iterative process undertaken during collation of studies identified using the search strategy

	Inclusion criteria	Exclusion criteria
**Population**	Surgery	Medical student
	Ophthalmology	Undergraduate
	Obstetrics and Gynaecology	Nursing and allied health
		Veterinary
		Non-surgical specialties
		Clerkship
**Context**	Must include a specific curriculum (or component thereof)	
**Concept**	Renewal	Development
	May include a specific focus on curricula renewal processes	Sources not focusing on curricula renewal (eg sources with a suggestion for renewal that is gratuitous
**Source characteristics**	Publication year 2003 – current	Publication year prior to 2003
		Abstract only

Appendix 3 – Included sources

**Author and date of publication**	**Publication title**	**Journal**
Sachdeva 2007 [36]	National efforts to reform residency education in surgery	Academic Medicine
Lillevang 2009 [53]	Evaluation of a national process of reforming curricula in postgraduate medical education	Medical Teacher
Webb 2009 [37]	Protected block curriculum enhances learning during general surgery residency training	Archives of Surgery
Moalem 2012 [38]	Three-phase model for surgical training: a proposal for improved resident training, assessment, and satisfaction	Journal of Surgical Education
Tapia 2014 [39]	Identifying and Eliminating Deficiencies in the General Surgery Resident Core Competency Curriculum	JAMA Surgery
Mansouri 2015 [40]	A Practical Methodological Approach Towards Identifying Core Competencies in Medical Education Based on Literature Trends: A Feasibility Study Based on Vestibular Schwannoma Science	Neurosurgery
Drolet 2017 [41]	Program Director Perceptions of the General Surgery Milestones Project	Journal of Surgical Education
Conforti 2018 [42]	The Effect and Use of Milestones in the Assessment of Neurological Surgery Residents and Residency Programs	Journal of Surgical Education
Nousiainen 2018 [43]	Eight-year outcomes of a competency-based residency training program in orthopedic surgery	Medical Teacher
Garbarino 2019 [44]	Current Trends in Psychiatric Education Among Obstetrics and Gynecology Residency Programs	Academic Psychiatry
Mador 2020 [45]	Development of a novel conceptual framework for curriculum design in Canadian postgraduate trauma training	Canadian Medical Education Journal
Incoll 2020 [52]	Australian orthopaedic surgery training: Australian Orthopaedic Association's strategic education review	ANZ Journal of Surgery
Gadjradj 2020 [50]	The neurosurgical curriculum: Which procedures are essential?	Interdisciplinary Neurosurgery

Dickinson 2020 [46]	Institution-specific utilization of the American College of Surgeons/Association of Program Directors operative skills curriculum: From needs assessment to implementation	Surgery
Chan 2021 [47]	A mixed methods evaluation of a 4-week geriatrics curriculum in strengthening knowledge and comfort among orthopaedic surgery residents	BMC Medical Education
Kearney 2022 [48]	The Accreditation Council for Graduate Medical Education Milestones in Integrated Plastic Surgery Programs: How Competency-Based Assessment Has Been Implemented	Plastic and Reconstructive Surgery
Dada 2022 [51]	State of African Neurosurgical Education: An Analysis of Publicly Available Curricula	World Neurosurgery
Ryan 2024 [49]	Redefining Trauma Training in Canada: A National Delphi Study on Curriculum, Educational Resources, and Training Initiatives	Journal of Surgical Education
